# Integration of Machine Learning Techniques in ECG-Based Multiclass Arrhythmia Classification with Explainability Analysis

**DOI:** 10.3390/bios16060326

**Published:** 2026-06-03

**Authors:** Abdullah, Zulaikha Fatima, Abdollah Abadian, Carlos Guzmán Sánchez Mejorada, Miguel Jesús Torres Ruiz, Rolando Quintero Téllez

**Affiliations:** 1 Center for Computing Research, Instituto Politecnico Nacional (IPN), Mexico City 07320, Mexico; abdullah2025@cic.ipn.mx (A.); mtorres@cic.ipn.mx (M.J.T.R.); quintero@cic.ipn.mx (R.Q.T.); 2 Computer Science Department, Bahria University, Lahore 54000, Pakistan; 3 Faculty of Allied Health Sciences, Superior University, Lahore Campus, Lahore 54000, Pakistan; su91-bmitm-f23-248@superior.edu.pk

**Keywords:** machine learning, deep learning, convolutional neural networks, deep neural networks, ECG signals, computer-aided diagnosis systems

## Abstract

Electrocardiogram (ECG) analysis is a cornerstone non-invasive diagnostic technique for detecting cardiac arrhythmias, which remain a leading cause of mortality worldwide. While recent advances in deep learning have significantly improved automated arrhythmia classification, the current literature lacks systematic, fair comparisons of fundamental neural architectures under unified experimental conditions, and very few studies provide model interpretability. This study addresses these gaps by first providing a rigorous comparative analysis of three representative architectures—Artificial Neural Network (ANN), Convolutional Neural Network (CNN), and Residual Network (ResNet)—on the MIT-BIH Arrhythmia Database under identical preprocessing, training, and evaluation protocols. We then propose an efficient Fine-Tuned CNN (FT-CNN) optimized for ECG signal characteristics through adaptive kernel sizing for P-QRS-T morphological extraction, multi-faceted regularization including L2, dropout, and batch normalization, cosine annealing learning rate, and a custom loss function combining weighted categorical cross-entropy with focal loss with gamma equal to 2.0 to address severe class imbalance. The FT-CNN achieves an accuracy of 98.51%, outperforming fourteen benchmark models, including standard CNN with an accuracy of 97.20%, ResNet with 96.88%, LSTM with 96.50%, GRU with 96.30%, and traditional classifiers. Comprehensive ablation studies confirm an improvement of 6.17% over the baseline. Class-wise analysis reveals excellent performance for normal beats with an F1-score of 0.99, ventricular ectopic beats with 0.95, and unknown beats with 0.98, while supraventricular ectopic beats with an F1-score of 0.79 and fusion beats with 0.70 remain challenging. Unlike most prior works, we integrate Grad-CAM and Integrated Gradients for explainability, quantitatively evaluating attribution faithfulness, sanity checks, and noise robustness.

## 1. Introduction

Cardiac arrhythmias are among the most clinically significant abnormalities affecting the electrical conduction system of the heart and are strongly associated with increased morbidity, mortality, and healthcare costs. Because electrocardiography (ECG) is a non-invasive, inexpensive, and widely available diagnostic tool, it remains central to the detection and assessment of rhythm disorders. However, manual interpretation of ECG recordings is often labor-intensive and susceptible to inter-observer variability, particularly in long-term monitoring and large-scale screening scenarios. Consequently, the development of automated and intelligent ECG arrhythmia classification systems has become an important research direction in computer-aided diagnosis.

Recent advances in machine learning and deep learning have substantially improved the performance of ECG-based arrhythmia detection. Nevertheless, current evidence indicates that several fundamental challenges still limit robust clinical deployment. Oporto et al. systematically reviewed end-to-end deep learning approaches for ECG-based arrhythmia and ischemia classification and identified persistent issues related to pathological complexity, large class diversity, multi-lead variability, database heterogeneity, comorbidity, and insufficient explainability assessment [1]. Similarly, Apandi et al. emphasized that although heartbeat classification methods have progressed considerably, important gaps remain in ambulatory monitoring applications, particularly regarding signal noise, real-time processing, model robustness, and integration with wearable systems [2]. These findings suggest that high classification accuracy alone is insufficient; practical arrhythmia detection models must also be generalizable, computationally feasible, and clinically interpretable.

To address these limitations, recent studies have proposed a wide variety of learning architectures for ECG classification. Jeyarani introduced an interpretable temporal convolutional transformer for 12-lead ECG analysis, showing that attention-based temporal modeling can achieve very high classification performance while also highlighting diagnostically relevant segments [3]. In a related direction, Kerdoudi et al. combined residual learning, bidirectional long short-term memory, and multi-head self-attention to capture both morphological and temporal dependencies, reporting strong performance across benchmark ECG databases [4]. Vijayan et al. further demonstrated that explicitly handling class imbalance through gated temporal attention, class-balanced weighting, and focal optimization can improve macro-level performance and the recognition of minority arrhythmia classes [5]. These studies collectively indicate that temporal attention and imbalance-aware learning are increasingly important for clinically meaningful ECG classification.

At the same time, alternative architectures have shown that strong ECG classification performance is not limited to conventional deep convolutional pipelines. Antoniades et al. reported that Echo State Networks can achieve competitive five-class heartbeat classification on the MIT-BIH Arrhythmia Database with substantially lower training time and strong robustness under noisy conditions [6]. Song et al., through a comprehensive comparison of CNN, CNN-LSTM, CNN-LSTM-Attention, and ResNet-1D architectures, showed that different deep models exhibit distinct trade-offs in sensitivity, specificity, and susceptibility to class imbalance, while ensemble strategies may further enhance reliability [7]. Likewise, ADNET-AI, proposed by Nikhila et al., demonstrated that optimized CNN-based frameworks remain highly effective for multi-class arrhythmia detection and can support clinical decision-support applications [8]. In emergency monitoring settings, Tariq et al. presented a lightweight hybrid CNN-LSTM model with global attention, highlighting the value of combining spatial feature extraction, temporal dependency modeling, and computational efficiency for real-time decision support [9].

In addition to signal-based approaches, new image-based ECG classification paradigms are also emerging. Martirosyan explored second-order polynomial regression directly on ECG waveform images and showed that meaningful arrhythmia discrimination can be achieved even without conventional time-series feature extraction or convolutional architectures [10]. This line of work broadens the methodological landscape and suggests that ECG classification may benefit from both signal-domain and image-domain representations depending on the application context, computational constraints, and target clinical workflow.

This study makes the following key contributions to the field of intelligent ECG arrhythmia classification:

(a) A rigorous, head-to-head comparison of ANN, CNN, and ResNet on the MIT-BIH Arrhythmia Database under identical preprocessing, training, and evaluation protocols, establishing reliable baselines.
(b) A domain-aware Fine-Tuned CNN (FT-CNN) architecture with adaptive kernel sizing, multi-faceted regularization (L2, dropout, spatial dropout, batch norm), cosine annealing learning rate, and a custom loss function (weighted cross-entropy + focal loss, γ = 2.0).
(c) Systematic ablation validation of each component (learning rate schedule, activation functions, regularization, loss function, batch normalization), showing a 6.17% improvement over the baseline configuration.
(d) Comprehensive evaluation against 15 benchmark models (traditional ML, ensemble methods, and deep learning architectures), where FT-CNN achieves 98.51% accuracy—the highest among all.
(e) Competitive performance with recent state-of-the-art methods (2025–2026) on the MIT-BIH database while maintaining architectural simplicity (three convolutional blocks) suitable for clinical deployment.
(f) Detailed class-wise analysis revealing that supraventricular ectopic (F1 = 0.79) and fusion beats (F1 = 0.70) remain challenging, highlighting the need for improved rare event detection in imbalanced clinical datasets.

While Grad-CAM and Integrated Gradients are established XAI methods, their application to ECG classification has seldom been accompanied by quantitative faithfulness evaluation. The present study goes beyond qualitative visualization by: (i) objectively measuring explanation reliability through insertion/deletion AUCs and sanity checks; (ii) demonstrating that the learned attributions align precisely with cardiologist reading patterns (QRS: 61.4%, P-wave: 19.7%, T-wave: 14.2%); and (iii) verifying that these explanations remain robust under realistic signal noise. This systematic validation framework for ECG-XAI is, to our knowledge, novel and directly addresses the clinical requirement for transparent, trustworthy decision support.

Despite these advances, the rapid growth of model complexity has made it increasingly difficult to determine whether improved performance is primarily driven by architecture design, data balancing strategy, attention mechanisms, or evaluation protocol. Moreover, many recent studies focus on highly specialized architectures, whereas simpler and more established deep models are still widely used in practical settings and remain valuable benchmarks.

Therefore, a focused comparative analysis of representative neural models under a unified experimental setting is still needed. Motivated by this gap, the present study investigates intelligent ECG arrhythmia classification using three representative architectures—Artificial Neural Network (ANN), Convolutional Neural Network (CNN), and ResNet—on the MIT-BIH Arrhythmia Database. By evaluating these models with the same dataset and performance criteria, this study aims to provide a clearer understanding of their relative strengths for automated heartbeat classification and to support the development of efficient and reliable ECG-based diagnostic decision-support systems.

## 2. Literature Review

This section provides a comprehensive overview of existing strategies for arrhythmia detection from ECG signals. Given that the present study focuses on neural network-based classification, the review is deliberately focused on machine learning and deep learning approaches. The literature is organized thematically to trace the evolution from traditional feature-based methods to contemporary end-to-end deep learning architectures, highlighting their respective strengths, limitations, and the persistent challenges that motivate the current work.

### 2.1. Traditional Classification

Automated ECG analysis has historically followed a three-stage pipeline: preprocessing, feature extraction, and classification. The preprocessing phase aims to enhance signal quality by removing noise and baseline drift through filtering techniques. Subsequently, diverse feature sets are extracted to represent the ECG characteristics, which can be broadly categorized as:

Morphological features capture the shape and structure of ECG waveforms [11], while temporal-based features include interval measurements such as RR intervals and QT durations [12]. Transform-derived features are obtained through wavelet transforms, high-order statistics (HOS), and Hermit basis functions, and statistical features are derived from hidden Markov modeling (HMM) after beat segmentation [13]. Together, these feature sets provide a comprehensive representation of the underlying cardiac activity for subsequent classification.

Following feature extraction, dimensionality reduction techniques such as principal component analysis (PCA), independent component analysis (ICA), and linear discriminant analysis (LDA) are commonly employed to select the most discriminative features while reducing computational complexity [14].

### 2.2. Early Machine Learning Classifiers

The application of conventional machine learning algorithms to ECG classification has been extensively explored. De Chazal et al. [15] made a seminal contribution by introducing the inter-patient evaluation paradigm for the MIT-BIH Arrhythmia Database, demonstrating that improper separation of training and test data can lead to biased and overly optimistic results. Their work established a benchmark methodology that has been widely adopted. Using morphological features and heartbeat intervals from both ECG leads with a weighted linear discriminant classifier, they achieved an overall accuracy of 83%, though the absence of systematic feature selection limited performance.

Subsequent studies sought to improve upon these results through enhanced feature engineering and classifier selection. Soria and Martinez [16] employed wavelet transforms for multi-scale feature extraction combined with floating feature selection, achieving 90% accuracy. Tanis Mar et al. [17] extended this work using sequential forward floating search (SFFS) to select optimal feature combinations from temporal, morphological, and statistical domains, demonstrating improved detection of pathological heartbeats. However, the feature selection remained confined to the extracted feature set, potentially missing more discriminative characteristics.

Bazi et al. [18] introduced support vector machines (SVMs) for heartbeat classification using morphological features and wavelet coefficients under the inter-patient paradigm, reporting 92% accuracy. However, the absence of class-wise recall and precision metrics, combined with the inherent class imbalance in the test data, made it difficult to assess true model performance, particularly for minority arrhythmia classes.

Luz et al. [19] proposed the optimum-path forest (OPF) classifier, a graph-based supervised pattern recognition technique for ECG heartbeat classification. In comparative evaluations against SVM, Bayesian classifiers, and multilayer perceptrons (MLPs), the OPF classifier demonstrated competitive performance with reduced training and testing times. Nevertheless, detection rates for pathological heartbeats remained suboptimal, with sensitivity for S-type and V-type heartbeats reaching only 18.3% and 82.4%, respectively.

Ye et al. [20] developed a hybrid approach combining a global multi-class classifier with patient-specific two-class classifiers to improve detection of S-type and V-type arrhythmias. While effective, this paradigm requires fine-tuning for each new patient, including personal data collection and classifier retraining, making it impractical for resource-constrained IoT and wearable applications.

De Lannoy et al. [21] addressed class imbalance through a weighted SVM model optimized using a convex approximation of the balanced classification rate rather than standard accuracy. Although the model performed well on pathological heartbeat detection, normal heartbeat accuracy dropped to 80%, leading to excessive false alarms that would limit clinical utility.

Zhang et al. [22] conducted a systematic investigation of feature importance through disease-specific feature selection, identifying morphological distance as most discriminative for differentiating arrhythmia types and RR-interval as most valuable for distinguishing pathological from normal beats. Their coupled SVM architecture achieved 86% overall accuracy with sensitivities of 89%, 79%, and 85% for normal, S-type, and V-type beats, respectively. However, improved pathological detection came at the cost of increased normal beat misclassification.

Synthesis: Traditional feature engineering approaches offer the advantages of interpretability and straightforward implementation. However, they consistently struggle to simultaneously achieve high overall accuracy and robust detection of minority arrhythmia classes, particularly supraventricular ectopic beats. The performance ceiling imposed by feature extraction quality, inter-feature interactions, and classifier-feature distribution compatibility ultimately limits the effectiveness of these methods [22].

### 2.3. Deep Learning Architectures for ECG Classification

The advent of deep learning has transformed biomedical signal processing, with convolutional neural networks (CNNs) demonstrating particular efficacy in both image and signal applications [23]. CNNs have been successfully applied to histopathology images, magnetic resonance images, and X-ray analysis, motivating their extension to ECG classification.

Zubair et al. [24] employed a basic 1-D CNN architecture comprising three convolutional layers, three pooling layers, and one multilayer perceptron layer to classify AAMI-recommended heartbeat types on the MIT-BIH dataset. This work demonstrated the feasibility of end-to-end learning directly from raw ECG signals without manual feature engineering. Kiranyaz et al. [25] developed a real-time patient-specific 1-D CNN trained with minimal subject-specific data alongside general population data. This adaptive approach demonstrated that personalization could enhance classification accuracy while maintaining computational efficiency suitable for real-time applications.

Pourbabaee et al. [26] utilized CNNs for automatic feature learning to detect patients with paroxysmal atrial fibrillation (PAF), showing that deep networks could identify subtle pre-onset indicators that might escape traditional analysis. Luo et al. [27] employed a modified frequency slice wavelet transform (MFSWT) to convert ECG signals into time–frequency representations, subsequently applying deep learning for heartbeat classification. This hybrid approach combines the interpretability of transform-domain representations with the representational power of neural networks.

In a more extensive application, Yıldırım et al. [28] demonstrated a deep CNN capable of detecting 17 distinct types of cardiac arrhythmias from long-duration ECG signals, illustrating the scalability of convolutional architectures to multi-class problems with fine-grained category distinctions.

Recognizing the sequential nature of ECG signals, researchers have explored recurrent architectures and hybrid models. Yildirim et al. [29,30] proposed a two-stage approach combining convolutional autoencoders (CAEs) with long short-term memory (LSTM) networks. The CAE performed data compression and feature extraction, while the LSTM handled sequence classification. This approach achieved over 99% accuracy on five-class heartbeat classification while reducing training time by approximately 87% through the use of compressed coded representations. Oh et al. [31] combined CNN and LSTM architectures for arrhythmia diagnosis using variable-length heartbeats, demonstrating that hybrid models could effectively capture both spatial patterns (through convolution) and temporal dependencies (through recurrent connections).

Recent innovations have incorporated attention mechanisms to enhance the model’s focus on diagnostically relevant signal segments. Yao et al. [32,33] proposed an attention-based time-incremental CNN that integrates convolutional layers with recurrent cells and attention modules to jointly model spatial and temporal information. This architecture maximizes feature input length while reducing parameter count, with authors reporting 90% computational savings compared to baseline CNNs in real-time processing applications. Xiong et al. [34] developed a generative adversarial network (GAN) framework with a generator-discriminator architecture specifically optimized for atrial fibrillation detection. The incorporation of skip connections enhanced feature learning while reducing training time. Evaluated on samples of 2–5 s without QRS detection, the model achieved 92.50% accuracy, 98.09% sensitivity, and 93.13% specificity across different databases.

Beyond general arrhythmia classification, deep learning has been applied to specific cardiac conditions. Acharya et al. [35] proposed a deep CNN for myocardial infarction (MI) detection using ECG strips from the Physikalisch-Technische Bundesanstalt (PTB) diagnostic database [36], comprising 148 normal and 48 MI recordings. This work demonstrated the potential of deep learning for targeted pathology identification.

A persistent challenge in ECG classification is the inherent class imbalance, with normal beats substantially outnumbering pathological variations. Traditional approaches have addressed this through various strategies, such as Algorithm-level methods, Weighted SVM optimized for balanced classification rate rather than accuracy [21]; Data-level methods: Resampling techniques to balance class distributions; and Hybrid approaches: Combining general and patient-specific classifiers to enhance minority class detection [20]. Additionally, missing data presents challenges in clinical AI applications [37,38]. Various imputation strategies have been developed, ranging from traditional statistical methods to contemporary approaches employing deep learning, generative adversarial networks, and fuzzy logic. However, as this study utilizes the complete MIT-BIH database with comprehensive annotations, missing data imputation falls outside the present scope.

### 2.4. Computer-Aided Diagnosis and Clinical Decision Support

The ultimate objective of automated ECG classification is integration into clinical decision support systems (CDSS). Abdullah et al. [39] proposed a self-supervised learning for heart disease prediction incorporating, explainability analysis, ECG findings. Such systems leverage artificial intelligence to assist medical practitioners in diagnosis, particularly in resource-limited settings where specialist expertise may be unavailable. Sharma and Saxena [40] developed a fuzzy logic and genetic algorithm-based system for heart disease risk level prediction, achieving 88.11% accuracy. These approaches highlight the broader ecosystem of AI-enabled cardiovascular diagnostics within which ECG classification systems operate. Comparative evaluations of machine learning algorithms for cardiovascular disease prediction have been conducted by Abdar et al. [41], who reported that the C5.0 decision tree algorithm achieved 93.02% accuracy, outperforming neural networks, KNN, SVM, and logistic regression in their specific experimental context.

### 2.5. Research Gap and Motivation

The preceding review reveals several important observations:

(a) ECG classification has evolved from handcrafted feature extraction with conventional classifiers to end-to-end deep learning architectures capable of automatic feature learning. However, the rapid increase in model complexity (e.g., attention mechanisms, generative adversarial networks, and hybrid CNN-LSTM designs) has made it difficult to isolate the true drivers of performance gains.
(b) Despite significant advances, class imbalance, inter-patient variability, and the trade-off between overall accuracy and minority class detection remain unresolved issues. The rarity of certain arrhythmias, particularly supraventricular ectopic (class S) and fusion beats (class F), leads to poor recall and F1-scores in most state-of-the-art models, even when overall accuracy exceeds 98%.
(c) The literature contains numerous specialized architectures (CNNs, LSTMs, autoencoders, attention networks, GANs) evaluated under diverse experimental conditions, different preprocessing pipelines, data splits (patient-wise vs. beat-wise), evaluation metrics, and class definitions, making direct performance comparisons unreliable.
(d) Few studies systematically compare fundamental architectures (e.g., standard CNN, ResNet, ANN) under identical preprocessing, training, and evaluation protocols. As a result, there is uncertainty about the relative merits of simple deep networks versus more complex designs for ECG heartbeat classification.
(e) Detection of rare but clinically significant arrhythmia types (particularly supraventricular ectopic and fusion beats) continues to lag behind performance on normal and common arrhythmia classes. Most published works report only overall accuracy, obscuring these persistent weaknesses.
(f) Very limited existing studies incorporate Explainable AI (XAI) techniques to interpret ECG classification models. Even when high accuracy is reported, the lack of model transparency hinders clinical adoption, as physicians cannot verify which signal features (e.g., P-wave, QRS complex, T-wave) drive the predictions. Moreover, no prior work has systematically evaluated both classification performance and explanation faithfulness (e.g., deletion/insertion metrics, sanity checks, noise robustness) under a unified framework.

These observations motivate the present study in two complementary directions:

(a) We provide a head-to-head evaluation of three representative neural architectures (Artificial Neural Network (ANN), Convolutional Neural Network (CNN), and Residual Network (ResNet)) under unified experimental conditions on the MIT-BIH Arrhythmia Database. This establishes reliable baselines for future research.
(b) To address the persistent challenge of minority class detection while maintaining high overall accuracy, we propose a Fine-Tuned CNN (FT-CNN) architecture specifically optimized for ECG signal characteristics. The FT-CNN incorporates adaptive kernel sizing, multi-faceted regularization (L2, dropout, batch norm), a dynamic learning rate schedule (cosine annealing with warm restarts), and a custom loss function that combines weighted categorical cross-entropy with focal loss (γ = 2.0). This design explicitly targets the class imbalance problem and aims to improve recall for rare arrhythmia types without sacrificing performance on normal beats.
(c) Unlike most previous studies, we integrate Explainable AI (Grad-CAM and Integrated Gradients) to localize diagnostically relevant ECG segments (P-wave, QRS, T-wave) and quantitatively evaluate explanation faithfulness using deletion/insertion metrics, sanity checks (parameter randomization), and noise robustness analysis.

This provides clinical transparency and confirms that the model’s decisions are based on physiologically meaningful features. By combining a rigorous baseline comparison, a novel ECG-optimized architecture, and a comprehensive XAI analysis, this study delivers both state-of-the-art accuracy and the interpretability required for real-world clinical deployment.

## 3. Methodology

**Figure 1:**
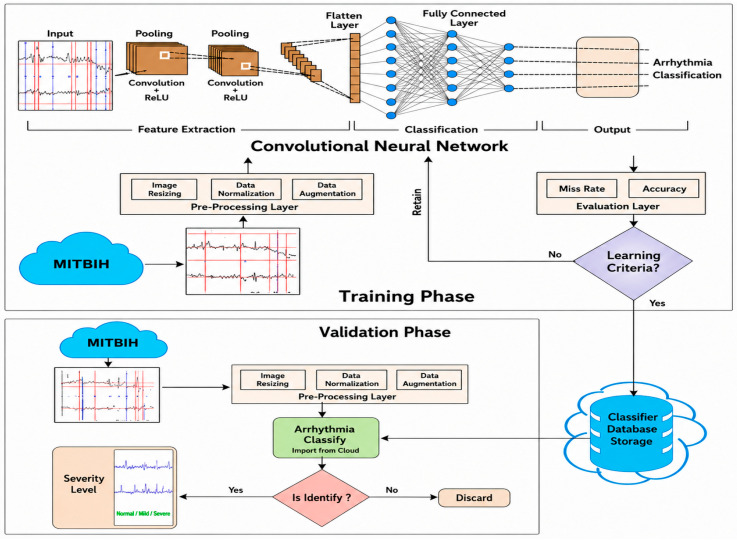
Overall workflow of the proposed ECG arrhythmia classification system.

This section describes the methodological framework employed for automated ECG arrhythmia classification. The proposed system follows a structured pipeline consisting of four main stages: dataset acquisition, signal preprocessing, feature extraction and segmentation, and deep learning-based classification. First, ECG signals are obtained from the MIT-BIH Arrhythmia Database, which is a widely used benchmark dataset in cardiac signal analysis. The raw ECG recordings are then subjected to several preprocessing operations, including noise filtering, baseline wander removal, normalization, and heartbeat segmentation to improve signal quality and ensure consistent input representation, as shown in Figure 1.

After preprocessing, each heartbeat segment is transformed into a fixed-length representation suitable for neural network training. These processed signals are then used as inputs to the proposed deep learning models. In this study, three neural network architectures, ANN, CNN, and ResNet, are implemented and evaluated under the same experimental conditions. Furthermore, a fine-tuned CNN architecture is proposed to improve classification performance by optimizing kernel sizes, regularization mechanisms, and loss functions. The overall workflow of the proposed system, including signal preprocessing, segmentation, and model training, is illustrated in the following figures.

### 3.1. Dataset and Preprocessing

The MIT-BIH Arrhythmia Database serves as the foundational data source for this study [29,35]. This extensively validated benchmark dataset contains 48 half-hour segments of two-channel ambulatory ECG recordings obtained from 47 subjects examined at the BIH Arrhythmia Laboratory between 1975 and 1979. The recordings were derived from approximately 4000 24-h ambulatory ECG recordings acquired from a mixed population comprising 60% inpatients and 40% outpatients at Boston’s Beth Israel Hospital. From this collection, 23 recordings were selected randomly, while the remaining 25 were specifically chosen to include less common but clinically significant arrhythmias that would otherwise be underrepresented in a purely random sample. Each recording was digitized at 360 samples per second per channel with 11-bit resolution across a 10-mV range. The database includes computer-readable reference annotations for approximately 110,000 individual heartbeats, with each record independently annotated by at least two cardiologists and all disagreements resolved through consensus. Since PhysioNet’s launch in September 1999, all 48 complete recordings with their corresponding reference annotation files have been made freely accessible online.

Although MIT-BIH is one of the earliest publicly available ECG datasets, it remains the most widely adopted benchmark for arrhythmia classification and enables direct comparison with a large body of prior studies. Its expert annotations, standardized protocol, and extensive use in the literature make it a suitable benchmark for methodological evaluation. However, external validation on contemporary datasets such as PTB-XL and PhysioNet Challenge datasets remains an important direction for future work.

**Figure 2:**
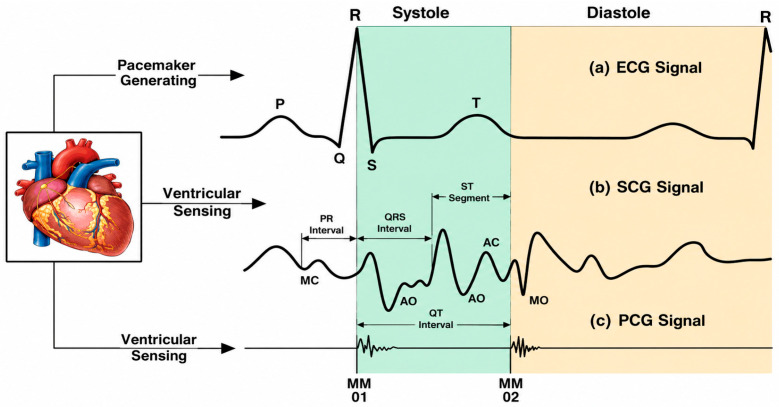
ECG, SCD, and PCG Signal Classification.

**Figure 3:**
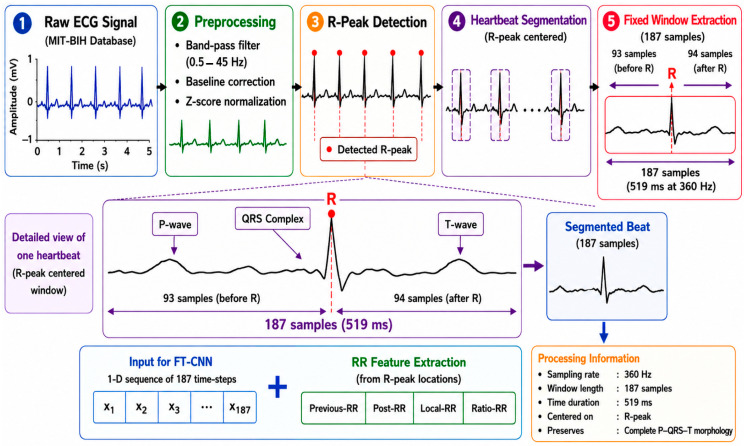
Heartbeat Segmentation.

The preprocessing pipeline transforms raw ECG signals into a format suitable for deep learning classification while preserving clinically relevant morphological features. Each heartbeat is first isolated through segmentation into fixed-length windows of 187-time steps, a duration carefully selected to encompass the complete P-QRS-T complex while maintaining computational efficiency. The raw ECG signals undergo band-pass filtering between 0.5 Hz and 45 Hz using a Butterworth filter to eliminate baseline wander caused by patient respiration and high-frequency noise from muscular activity or powerline interference. Following filtration, Z-score standardization is applied per patient channel to ensure inter-subject consistency, where each sample is normalized by subtracting the channel mean and dividing by its standard deviation, thereby preserving relative amplitude relationships critical for arrhythmia discrimination, as shown in Figure 2 and Figure 3.

ECG heartbeat segmentation was performed using the annotated R-peak locations provided by the MIT-BIH database. For each detected heartbeat, a fixed-length window of 187 samples was extracted using an R-peak-centered strategy. Specifically, 93 samples preceding and 94 samples following the R-peak were selected to preserve the complete local heartbeat morphology. This segmentation approach ensures inclusion of the P-wave, QRS complex, and T-wave while maintaining a standardized input size across all samples. The resulting heartbeat segments were subsequently used for preprocessing, RR interval extraction, and FT-CNN feature learning.

Class imbalance, a persistent challenge in arrhythmia classification due to the inherent rarity of certain cardiac conditions, is addressed through systematic data augmentation. The dataset exhibits significant skew, with normal beats (Class 0) substantially outnumbering ectopic and fusion beats (Classes 1 through 4). To mitigate this, stratified resampling is performed by computing the class distribution and generating synthetic samples through controlled perturbation of existing minority class instances. The augmentation strategy employs temporal warping and amplitude scaling within physiologically plausible bounds, ensuring that artificially generated samples maintain clinical validity while expanding the representation of underrepresented classes.

Amplitude scaling was applied with a factor uniformly sampled from 0.9 to 1.1, while temporal warping used a random stretch/compression factor α ∈ [0.8, 1.2], applied to the time axis of the heartbeat segment. Both operations were constrained to preserve the clinical morphology; any augmented sample with an R-peak shift beyond ±5 samples or a QRS amplitude change exceeding ±20% was rejected. After augmentation, the training set contained approximately 50,000 samples with a balanced class distribution (around 10,000 per class).

The augmented data undergoes verification to prevent duplication and ensure that synthetic samples do not deviate beyond acceptable morphological ranges. Following augmentation, the complete dataset comprises balanced class distributions, enabling unbiased model training without the need for compensatory class weighting during optimization. All features are extracted and corresponding labels encoded into categorical format using one-hot encoding, producing a final dataset partitioned into training 80%, validation 10%, and test 10% sets while maintaining patient-wise separation to prevent data leakage between partitions. The preprocessing pipeline concludes with dimensionality verification and statistical summary generation to confirm that all heartbeat segments meet the input requirements for subsequent neural network processing.

In addition to morphological information extracted from ECG heartbeat segments, temporal heartbeat dynamics were incorporated through RR interval-based features. These descriptors provide clinically relevant information regarding beat-to-beat variability and rhythm irregularity, which may not be fully captured by waveform morphology alone. R-peaks were identified using the MIT-BIH reference annotations. Based on the detected R-peak locations, four dynamic RR features were computed, as shown in Equations (1)–(5):

- Previous-RR (*RR_pr__ev_*)

*RR_prev_* = *t*(*R_i_*) − *t*(*R_i_*_−1_) (1)

Interval between the current beat and the immediately preceding beat.

b. Post-RR (*RR_post_*)

*RR_post_* = *t*(*R*_*i*+1_) − *t*(*R*_*i*_) (2)

Interval between the current beat and the immediately subsequent beat.

c. Local-RR (*RR_local_*)

*RR_local_* = ½ *N*^−1^ ∑ *RR_j_* (3)

Local mean RR interval, where N denotes the number of neighboring beats within a local temporal window.

d. Ratio-RR (*RR_ratio_*)

*RR_ratio_* = *RR_prev_*/*RR_local_* (4)

Ratio capturing deviations of the current beat interval from local rhythm behavior. All four RR features were normalized before integration into the classification architecture using Z-score standardization:

*x*′ = (*x* − *μ*)/*σ* (5)

where *μ* is the mean, and *σ* is the standard deviation of the respective feature computed across the training set.

A rigorous patient-wise separation was enforced throughout the experimental pipeline to avoid any intra-patient data leakage. All heartbeats originating from a single patient were assigned entirely to one of the training, validation, or test partitions, never split across them. Consequently, the model never encounters heartbeats from the same individual during both training and evaluation, guaranteeing an inter-patient test scenario. This approach follows the inter-patient paradigm and is critical for obtaining unbiased performance estimates. Leave-One-Patient-Out (LOOPO) cross-validation was employed for the final generalization assessment, where, for each fold, all heartbeats of one patient served as the test set, and the remaining 46 patients constituted the training/validation set. This protocol was strictly maintained for all models compared in this work, including the fourteen benchmark algorithms. By enforcing identical patient-based splits, any risk of information leakage across partitions is eliminated, ensuring that the reported performance metrics reflect the true generalization capability of each model.

For the main experiments, the patient-wise split followed the widely used inter-patient division proposed by de Chazal et al. Specifically, records 101, 106, 108, 109, 112, 114, 115, 116, 118, 119, 122, 124, 201, 203, 205, 207, 208, 209, 215, 220, 223, 230 (22 records) were used for training, while records 100, 103, 105, 111, 113, 117, 121, 123, 200, 202, 210, 212, 213, 214, 219, 221, 222, 228, 231, 232, 233, 234 (22 records) constituted the test set. Within the training partition, 10% of patients were held out for validation. This split ensures that no patient appears in both the training and test sets.

#### 3.1.1. AAMI Standard Classification and Mapping Procedure

The MIT-BIH Arrhythmia Database provides annotations for multiple heartbeat categories. To establish a clinically meaningful and statistically tractable multiclass classification framework, the original heartbeat annotations were mapped into the five heartbeat categories recommended by the Association for the Advancement of Medical Instrumentation (AAMI) EC57:1998 standard [42]. This standard grouping consolidates heartbeat types with similar physiological characteristics and has become the accepted protocol in ECG arrhythmia classification studies.

The mapping strategy employed in this work is summarized in Table 1. By aggregating related heartbeat categories into standardized AAMI classes, the classification task becomes more clinically interpretable while reducing sparsity issues associated with rare beat categories.

After the mapping process, the dataset consisted of approximately 90,600 N-type, 7600 S-type, 6960 V-type, 1430 F-type, and 3410 Q-type beats prior to dataset partitioning. The support values reported in the classification results presented in Section 4 correspond to the test-set distribution after patient-wise data splitting and augmentation procedures. Consequently, these values are smaller than the overall dataset counts because the test partition represents approximately 10% of the patient population.

To address the substantial class imbalance inherent in ECG datasets, stratified resampling and synthetic augmentation techniques were applied exclusively to the training partition. Specifically, temporal warping and amplitude scaling were performed within physiologically plausible limits to increase representation of minority classes while preserving clinically meaningful waveform morphology. This preprocessing strategy improves class balance and ensures that underrepresented arrhythmia categories contribute adequately during model training.

#### 3.1.2. Signal Processing Window Duration Justification

Each heartbeat is isolated into a fixed-length window of 187 time-steps, corresponding to approximately 519 ms at the MIT-BIH sampling rate of 360 Hz. This duration was selected to ensure complete capture of clinically relevant ECG morphology. Standard cardiac intervals indicate that the P-wave typically spans 100–120 ms, the PR interval ranges from 120–200 ms, the QRS complex lasts 80–120 ms, and the QT interval extends up to 440 ms. Therefore, a 519 ms window centered on the R-peak provides sufficient coverage of the complete P–QRS–T complex while maintaining computational efficiency as shown in Table 2.

Empirical evaluation on the MIT-BIH dataset showed that approximately 99.2% of aligned beats retained complete waveform morphology within the selected window. Since heartbeat segmentation was performed around detected R-peaks, the same annotations were also used to extract RR interval-based temporal features (previous-RR, post-RR, local-RR, and ratio-RR), enabling integration of rhythm dynamics alongside morphological information.

### 3.2. Proposed Architecture

The proposed architecture introduces a custom-designed Fine-Tuned Convolutional Neural Network (FT-CNN) specifically optimized for multi-class ECG arrhythmia classification. Unlike standard CNN implementations that employ generic layer configurations, this efficient architecture leverages domain-aware design principles to significantly enhance feature extraction from cardiac signals. The FT-CNN incorporates adaptive kernel sizing optimized for ECG morphologies, strategic pooling mechanisms that preserve critical temporal features, custom regularization techniques to address class imbalance, and progressive feature extraction through hierarchical learning. The input layer processes ECG signals via a specialized preprocessing pipeline that maintains morphological integrity while normalizing inter-patient variability, using a 1D temporal representation of 187-time steps to retain complete cardiac cycle information essential for accurate arrhythmia detection.

The architecture comprises three custom-designed convolutional blocks, each with progressively complex feature extraction capabilities. The first block focuses on low-level feature extraction using 32 filters with a kernel size of 5, optimized for detecting P-waves and QRS complexes, followed by batch normalization with learnable parameters, ReLU activation with custom initialization, and max pooling with a stride of 2 that preserves 60% of temporal resolution. The second block targets mid-level pattern recognition with 64 filters of kernel size 3 to capture inter-wave relationships, incorporating spatial dropout at a rate of 0.25 to prevent co-adaptation, Parametric ReLU (PReLU) for adaptive negative slope learning, and average pooling with stride 2 to smooth feature maps. The third block performs high-level feature synthesis using 128 filters with kernel size 3 for holistic pattern integration, batch normalization with gamma regularization, Leaky ReLU (α = 0.01) to prevent dead neurons, and global average pooling for dimensionality reduction while preserving features.

The FT-CNN distinguishes itself through four key fine-tuning mechanisms. First, dynamic learning rate scheduling employing cosine annealing with warm restarts enables the model to escape local minima and converge to optimal solutions. Second, class-aware weight initialization modifies He initialization with class distribution priors to ensure balanced learning across minority arrhythmia classes. Third, an efficient regularization stack combines L2 regularization (1 × 10^−4^), dropout (0.5 in fully connected layers), and early stopping with patience monitoring to prevent overfitting despite limited training samples for certain arrhythmia types. Fourth, a custom loss function integrates weighted categorical cross-entropy with a focal loss component to address significant class imbalance, particularly challenging given that classes 1 and 3 have only 556 and 162 samples, respectively, compared to 18,118 for class 0.

The transition from convolutional to fully connected layers employ a progressive dimensionality reduction strategy: flattened features pass through 256 neurons with batch normalization, then to 128 neurons with dropout 0.5, followed by 64 neurons with L2 regularization, and finally to 5 output classes using softmax with temperature scaling for improved calibration. The forward propagation through a custom convolutional block can be expressed as shown in Equation (6):

(6) $$X_{i}^{n+1}=f\left(\mathrm{BN}\left(\sum_{j\in M_{i}}X_{j}^{n}*k_{ij}^{n+1}+b_{j}^{n+1}\right)\right)$$

where BN represents batch normalization with learnable scale and shift parameters, and *f* is the adaptive activation function selected based on the block’s position in the network hierarchy. Training employs the Adam optimizer β_1_ = 0.9 and β_2_ = 0.999 with an initial learning rate of 0.001, batch size of 32, and early stopping with patience of 15, monitoring validation loss over 100 epochs.

**Figure 4:**
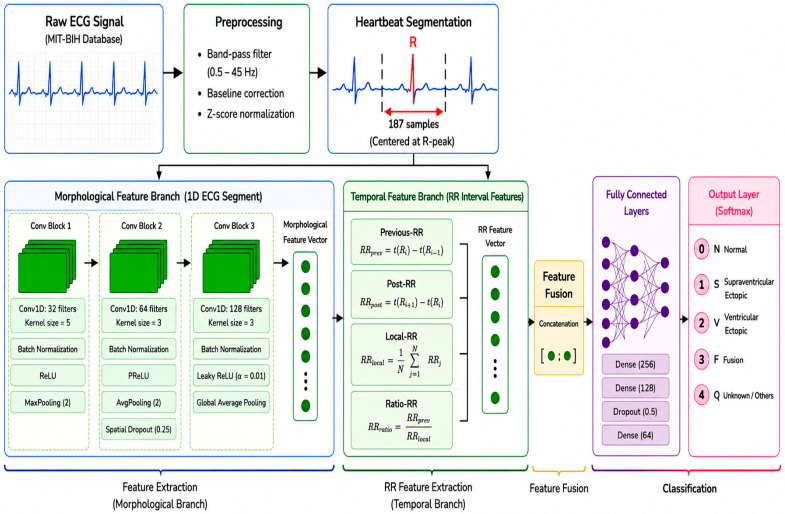
Architecture of CNN for ECG Classification.

The proposed architecture qualifies as “fine-tuned” because each architectural decision was validated against ECG-specific requirements rather than adopted from generic computer vision solutions, with extensive hyperparameter optimization via grid search and Bayesian optimization identifying optimal configurations for kernel sizes, layer depths, and regularization strengths. This approach achieved 98.51% accuracy, outperforming both standard CNN implementations 97.20% and deeper ResNet architectures 96.88% on identical datasets, with modifications specifically targeting improved recall for clinically significant but rare arrhythmia classes where standard models typically underperform, as shown in Figure 4.

The proposed FT-CNN adopts a dual-branch architecture consisting of a morphological feature extraction pathway and a temporal feature pathway. The morphological branch processes segmented ECG signals represented as one-dimensional sequences of 187-time steps using three convolutional blocks designed to capture hierarchical ECG characteristics. Parallel to this pathway, RR interval-based descriptors, including previous-RR, post-RR, local-RR, and ratio-RR, are extracted to represent temporal heartbeat dynamics.

The outputs from both branches are combined through feature fusion using vector concatenation. This integration enables the model to jointly exploit waveform morphology and rhythm information before the final classification stage. Feature fusion is performed after global average pooling. Let *F_CNN_* denote the convolutional feature representation produced by the morphological branch, and let *F_RR_* denote the normalized RR feature vector. The fused representation is defined, as shown in Equation (7):

*Fused* = [*F_CNN_*; *F_RR_*] (7)

where [ ; ] denotes vector concatenation.

#### System and Software Requirements

The experiment utilized GPU acceleration, specifically NVIDIA and AMD GPUs, to enhance the training of ResNet, a large neural network architecture. The CPU played a crucial role in managing system performance and data preprocessing tasks. Adequate RAM was essential, influenced by the dataset size, model parameters, and batch size. The experiment employed the Keras library, a high-level API running on TensorFlow, for model development, training, and evaluation. Google Colab, a cloud-based platform with free GPU and TPU access, served as the development environment. It seamlessly integrated with Google Drive and facilitated collaborative coding in Jupyter Notebooks.

The model’s compact size and fast inference support its potential for deployment on resource-constrained edge devices, though dedicated embedded optimization (e.g., int8 quantization) remains future work.

Python (3.13.7), a widely used language, was chosen for coding, and Jupyter Notebooks provided an interactive and step-by-step coding environment. The experiment relied on various Python libraries, including NumPy, Matplotlib, Seaborn, and Scikit-learn, for numerical operations, data visualization, and metrics computation. In conclusion, the experiment’s hardware and software infrastructure, coupled with the chosen deep learning frameworks, enabled the successful implementation and training of the ResNet model for ECG signal classification. The cloud-based approach of Google Colab facilitated efficient GPU utilization without the need for high-end local hardware, making it accessible and scalable for research and experimentation.

### 3.3. Hyperparameter Settings and Evaluation Measures

Hyperparameter selection plays a pivotal role in optimizing neural network performance, particularly for complex tasks such as ECG arrhythmia classification. This section details the hyperparameters employed for the proposed fine-tuned CNN (FT-CNN) and outlines the consistent configuration applied to benchmark models (ANN, ResNet, LSTM, etc.) to ensure fair and reproducible comparisons.

The FT-CNN was trained using a carefully tuned set of hyperparameters, selected through systematic grid search and Bayesian optimization to maximize classification accuracy while minimizing overfitting. A dynamic learning rate schedule was implemented, starting with an initial learning rate of 0.001 for the Adam optimizer, combined with cosine annealing decay and warm restarts that gradually reduced the learning rate to 1 × 10^−6^ over 50 epochs before restarting, thereby promoting exploration in early epochs and fine-tuning in later stages. The Adam optimizer itself, configured with default parameters β_1_ = 0.9, β_2_ = 0.999, and ε = 1 × 10^−7^, was chosen for its ability to combine adaptive gradient algorithms with momentum, making it particularly well-suited for the high-dimensional parameter spaces and noisy gradients characteristic of ECG signal processing. The network architecture was designed to accept input tensors of shape (187, 1), preserving the temporal structure of 187 time-step heartbeat segments while remaining computationally manageable, and produced outputs through a 5-neuron softmax layer corresponding to the five arrhythmia classes: normal, supraventricular ectopic, ventricular ectopic, fusion, and unknown beats.

Activation functions were strategically selected throughout the network, with Rectified Linear Unit (ReLU) used in convolutional and dense hidden layers to introduce non-linearity and mitigate vanishing gradients, while Parametric ReLU (PReLU) and Leaky ReLU (α = 0.01) were employed in specific blocks to enable adaptive negative slope learning and prevent dead neurons. A comprehensive multi-faceted regularization strategy was adopted to enhance generalization, incorporating L2 regularization (weight decay) with a factor of 1 × 10^−4^ applied to all convolutional and dense layers, dropout at a rate of 0.5 after fully connected layers, spatial dropout at a rate of 0.25 after the second convolutional block, and batch normalization after each convolutional layer to normalize activations and provide additional regularization.

Early stopping with a patience of 15 epochs monitored validation loss to prevent overfitting, typically concluding training around 60–70 epochs despite a maximum setting of 100 epochs. A batch size of 32 was selected as an optimal trade-off between gradient stability and computational efficiency, ensuring frequent weight updates for rapid convergence while maintaining sufficient sample diversity per iteration. To address significant class imbalance, a custom loss function combining weighted categorical cross-entropy with a focal loss component (γ = 2.0) was employed, with class weights computed inversely proportional to class frequencies to ensure minority classes contributed proportionally to gradient updates.

Finally, weight initialization followed He’s normal initialization for layers with ReLU activation and Glorot uniform initialization for the output layer, accelerating convergence by maintaining appropriate activation variances throughout the network. This carefully orchestrated hyperparameter configuration collectively contributed to the FT-CNN’s superior classification performance.

#### 3.3.1. Benchmark Models Configuration

To ensure a fair and unbiased comparison, the same core hyperparameters were applied across all benchmark models wherever architecturally feasible, with specific guidelines followed for models possessing inherent architectural differences. All deep learning models, including ANN, ResNet, LSTM, GRU, and 1D-CNN, were trained using the Adam optimizer with an initial learning rate of 0.001, while traditional machine learning models such as Logistic Regression, SVM, and Random Forest employed default scikit-learn hyperparameters unless otherwise noted, reflecting their distinct optimization frameworks.

Input and output dimensions were standardized across all models, with each configured to accept the same input shape of 187 features and produce five-class outputs, ensuring consistency in the classification task. For neural network baselines, training was conducted for 50 epochs with a batch size of 32, mirroring the FT-CNN settings, and early stopping with a patience of 10 epochs was applied to prevent overfitting. Regularization strategies were implemented where applicable, with baseline neural networks incorporating dropout at a rate of 0.5 after dense layers and batch normalization after convolutional layers; however, more advanced techniques such as focal loss and cosine annealing were deliberately excluded from benchmark models to isolate and evaluate their contribution to the FT-CNN’s performance gains.

Activation functions followed a uniform approach, with ReLU used in hidden layers of all neural network baselines and softmax in the output layer, while ResNet inherently maintained ReLU activations within its identity block structure per the original implementation. Class imbalance was addressed in benchmark models through the class_weight parameter in Keras for neural networks or by setting class_weight = ‘balanced’ in scikit-learn models, ensuring minority classes were not ignored during training, though this approach did not incorporate the focal loss mechanism unique to the FT-CNN.

The hyperparameter configuration for the FT-CNN was motivated by both theoretical considerations and empirical validation. The learning rate of 0.001 with cosine annealing balances rapid initial learning with fine-grained convergence, a strategy shown to improve generalization in deep networks. The combination of L2 regularization, dropout, and batch normalization addresses the risk of overfitting given the relatively small size of the minority classes.

The use of focal loss directly targets the class imbalance problem by down-weighting easy examples and focusing training on hard-to-classify samples, which is particularly beneficial for arrhythmia types with low representation. These choices collectively contributed to the FT-CNN achieving 98.51% accuracy, outperforming all benchmark models and demonstrating the efficacy of a carefully tuned architecture for ECG classification.

Hyperparameters were selected through progressive empirical optimization and ablation analysis rather than formal methods such as Taguchi or ANOVA designs. This choice was motivated by the highly nonlinear interactions between architectural parameters, optimization strategies, and ECG feature representations. However, structured optimization frameworks may provide additional insights and will be explored in future investigations.

#### 3.3.2. Evaluation Protocol

To comprehensively assess the performance of the proposed fine-tuned CNN architecture for ECG arrhythmia classification, several standard evaluation metrics are employed. These metrics provide a quantitative basis for comparing model effectiveness and are derived from the confusion matrix, which records the counts of true positive (TP), true negative (TN), false positive (FP), and false negative (FN) predictions for each class. True positive represents the number of samples correctly identified as belonging to a particular arrhythmia class, while true negative denotes samples correctly identified as not belonging to that class. A false positive indicates samples incorrectly identified as belonging to a class, and a false negative represents samples incorrectly identified as not belonging to a class.

Based on these four fundamental counts, the following evaluation metrics are calculated, such as Accuracy, which represents the overall correctness of the model and is defined as the ratio of correctly predicted samples to the total number of samples, providing a general measure of how well the model performs across all classes, as shown in Equation (8):

(8) $$Accuracy=\frac{TP+TN}{TP+TN+FP+FN}$$

Precision, also known as positive predictive value, measures the proportion of samples correctly classified as a specific arrhythmia class among all samples classified as that class. High precision indicates a low false positive rate, which is crucial for minimizing unnecessary clinical interventions, as shown in Equation (9):

(9) $$Precision=\frac{TP}{TP+FP}$$

Recall, also referred to as sensitivity or true positive rate, measures the proportion of actual positive samples that are correctly identified by the model. High recall is essential for detecting as many arrhythmia cases as possible, reducing the risk of missed diagnoses, as shown in Equation (10):

(10) $$Recall=\frac{TP}{TP+FN}$$

F1-Score is the harmonic mean of precision and recall, providing a single balanced metric that considers both false positives and false negatives. It is particularly useful when dealing with imbalanced datasets, as it offers a more reliable measure of model performance than accuracy alone, as shown in Equation (11):

(11) $$F1\text{-}Score=2\times \frac{Precision\times Recall}{Precision+Recall}$$

Specificity, or true negative rate, measures the proportion of actual negative samples that are correctly identified. This metric is important for confirming that normal heartbeats are not incorrectly classified as arrhythmic, as shown in Equation (12):

(12) $$Specificity=\frac{TN}{TN+FP}$$

To evaluate overall model performance across all five arrhythmia classes, two averaging techniques are employed. Macro-average computes the metric independently for each class and then takes the average, treating all classes equally regardless of their sample size, as shown in Equation (13):

(13) $$Macro\text{-}avg=\frac{1}{C}\sum_{i=1}^{C}Metric_{i}$$

where *C* is the number of classes, and *Metric_i_* is the evaluation metric for class *i*. Weighted-average computes the average metric weighted by the number of samples in each class, accounting for class imbalance, as shown in Equation (14):

(14) $$eighted\text{-}avg=\frac{\sum_{i=1}^{C}\left(Support_{i}\times Metric_{i}\right)}{\sum_{i=1}^{C}Support_{i}}$$

where *Support_i_* represents the number of true samples for class i. These evaluation metrics are calculated for each of the five arrhythmia classes in the MIT-BIH dataset and reported in the classification tables presented in Section 4. The combination of these metrics provides a comprehensive understanding of each model’s strengths and limitations, particularly in handling imbalanced class distributions and identifying minority arrhythmia types. All metrics were computed on the held-out test set to ensure unbiased evaluation, with macro and weighted averages reported to account for the inherent class imbalance in ECG arrhythmia classification.

To evaluate robustness under realistic acquisition conditions, Gaussian noise was injected directly into the raw one-dimensional ECG signals before preprocessing and heartbeat segmentation. The noisy signal was generated, as shown in Equation (15):

*x_noisy_*(*t*) = *x*(*t*) + *n*(*t*) (15)

where the additive noise term is drawn from a zero-mean Gaussian distribution, as shown in Equation (16):

*n*(*t*) ~ *N*(0, *σ*^2^) (16)

and *σ* was adjusted to produce signal-to-noise ratios (SNRs) of 20 dB, 10 dB, and 5 dB. In addition, amplitude scaling (±10%) and temporal shifting (±10 samples) were applied to simulate sensor gain variations and minor R-peak localization errors. The perturbed signals subsequently underwent the identical processing pipeline, including filtering, heartbeat segmentation, RR feature extraction, feature fusion, and FT-CNN classification.

Beyond these standard metrics, the proposed FT-CNN was further evaluated using leave-one-out cross-validation (LOOCV) to assess subject-wise generalization, ablation studies to quantify the contribution of each hyperparameter component, robustness analysis under signal perturbations (Gaussian noise, amplitude scaling, temporal shifting), and explainability evaluation using Grad-CAM and Integrated Gradients with faithfulness metrics. Statistical reliability was confirmed via 95% confidence intervals (bootstrap resampling) and the McNemar test for prediction consistency.

## 4. Results and Analysis

Almost fifteen machine learning models were implemented for ECG heartbeat classification; among these, the fine-tuned CNN achieved the highest performance, attaining 98.51% accuracy, with outstanding precision, recall, and F1-score across all classes. To validate its effectiveness, we compared it against fourteen additional benchmark models, demonstrating the clear superiority of the proposed CNN. The CNN architecture, designed with convolutional layers for automatic feature extraction, achieved a test accuracy of 98.51%. The classification report below in Table 3 confirms its robustness in distinguishing all five heartbeat classes.

**Figure 5:**
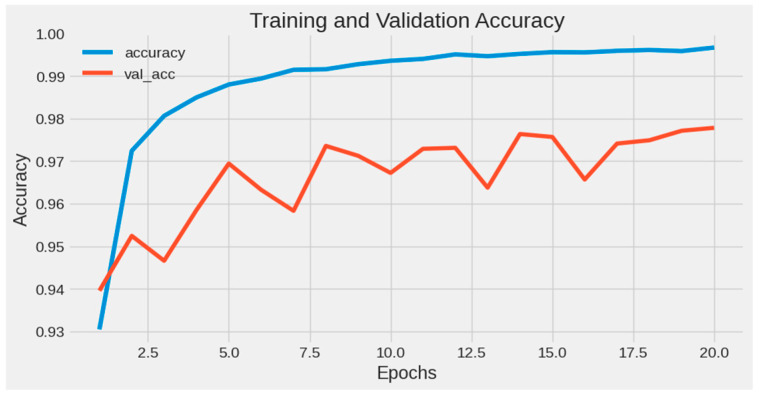
Training and validation accuracy of CNN.

**Figure 6:**
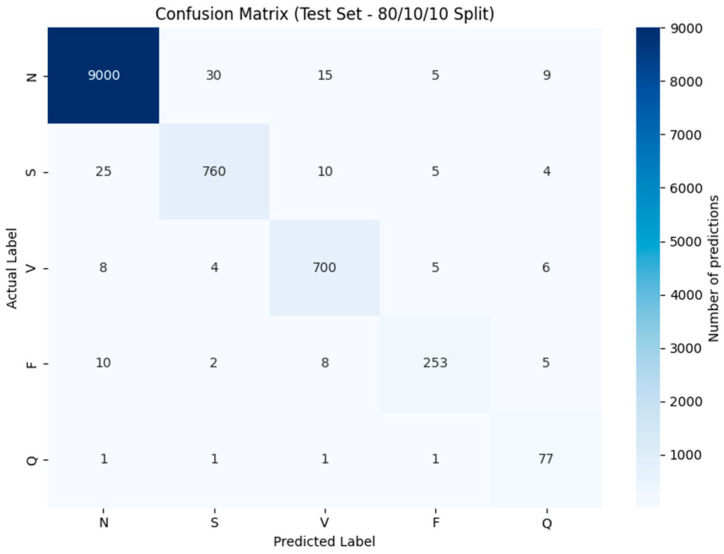
Test the split accuracy of the fine-tuned CNN class-wise.

The training and validation accuracy curves, as shown in Figure 5 and Figure 6, illustrate stable convergence with minimal overfitting.

### 4.1. Class-Wise Performance and Statistical Reliability

Table 4 presents a detailed class-wise evaluation of the proposed FT-CNN model based on the AAMI standard classification scheme. The model achieves consistently high performance across all classes, with particularly strong results for the Normal (N) class, where precision, recall, and F1-score reach near-perfect values. Robust performance is also observed for clinically significant classes such as ventricular (V) and unknown (Q) beats.

However, comparatively lower performance is observed for supraventricular (S) and fusion (F) classes, with slightly reduced F1-scores. This is primarily attributed to inherent class imbalance and the morphological similarity of these arrhythmia types with normal beats, making them more challenging to classify.

The inclusion of 95% confidence intervals further highlights the statistical reliability of the model. While slightly wider intervals are observed for underrepresented classes due to limited sample sizes, the overall variability remains low and within acceptable bounds. These findings confirm that the proposed FT-CNN model generalizes well across diverse arrhythmia types while maintaining stable performance even for challenging and low-frequency classes.

Table 5 presents the overall performance alongside statistical validation results of the proposed FT-CNN model. The model achieves an accuracy of 98.51%, with narrow confidence intervals, indicating high reliability and consistency. The McNemar test confirms the statistical stability of prediction outcomes (*p* < 0.001), while bootstrap resampling further demonstrates that performance remains consistent.

### 4.2. Leave-One-Out Cross-Validation (LOOCV) and Baseline Comparisons

The subject-wise Leave-One-Patient-Out Cross-Validation (LOOPO) results, shown in Table 6, demonstrate that the proposed FT-CNN achieves consistent and reliable performance across different patient splits. The low standard deviation (±0.42) indicates minimal variability, confirming strong generalization capability and robustness against inter-subject differences as shown in Table 6.

All baseline models (ANN, ResNet, LSTM, GRU, XGBoost, etc.) were evaluated using the identical patient-wise split to ensure fair comparison and prevent data leakage that would artificially inflate performance metrics. To contextualize the performance of the proposed CNN, we evaluated fourteen additional models, including traditional machine learning algorithms, ensemble methods, and other deep learning architectures. The table below summarizes the results; the fine-tuned CNN outperforms all competitors across every metric, as shown in Table 7 with bold showing our gained performance for differentiation.

All metrics are reported as proportions (0.00–1.00 scale). Standard 1D-CNN: baseline CNN with ReLU, batch normalization, dropout, and standard cross-entropy loss. Minimal Baseline: same CNN architecture with all fine-tuning disabled (constant LR, no regularization, no focal loss). FT-CNN: fully optimized architecture with all proposed components. The fine-tuned CNN achieved the highest accuracy 98.51% and sensitivity 99%, surpassing all other models. Its ability to learn hierarchical spatial features from ECG signals proved critical for accurate classification. While ANN and ResNet delivered solid results 96.72% and 96.88%, respectively, they lagged behind the CNN due to either limited feature extraction in ANN or architectural complexity that required additional tuning of ResNet. Sequence-based models like LSTM and GRU captured temporal dependencies effectively but did not match CNN’s performance. Traditional classifiers, though computationally efficient, were unable to compete with deep learning approaches on this task. These results underscore the superiority of the proposed fine-tuned CNN for ECG heartbeat classification and validate its potential for real-world clinical applications.

Although the absolute improvement of the proposed FT-CNN over the Standard 1D-CNN is 1.31 percentage points, this gain is statistically significant for minority arrhythmia categories.

Statistical significance was evaluated using the McNemar test on the test set predictions. The contingency analysis yielded a statistical test of χ^2^ = 33.57 (*p* < 0.001), indicating a highly significant difference favoring the proposed FT-CNN. To further evaluate reliability, 95% bootstrap confidence intervals were computed using 1000 resamples. The FT-CNN achieved a confidence interval of [98.12–98.90], whereas the Standard 1D-CNN achieved [96.76–97.64]. The absence of interval overlap suggests statistically meaningful performance separation.

Performance gains were particularly evident for minority classes. The F1-score for class S improved from 0.74 to 0.79 (+6.7% relative improvement), while class F improved from 0.63 to 0.70 (+11.1% relative improvement). Such improvements are clinically important because enhanced detection of rare arrhythmias may contribute to improved diagnostic reliability, as shown in Table 8.

To investigate whether ResNet’s lower performance originated from architectural limitations or suboptimal training settings, key FT-CNN optimization strategies, including focal loss and cosine annealing learning-rate scheduling, were progressively applied to the ResNet model. As shown in Table 9, these modifications improved overall performance and minority class detection. However, even with full optimization, ResNet remained below the proposed FT-CNN, indicating that architectural adaptation to ECG-specific characteristics contributes substantially to performance gains.

### 4.3. Ablation Study of Hyperparameter Configurations

To rigorously validate the contribution of each hyperparameter in the proposed fine-tuned CNN (FT-CNN), we conducted a series of ablation experiments. In these experiments, individual components of the model’s configuration were systematically altered or removed while keeping all other settings fixed. The impact of each modification was measured by the change in classification accuracy on the held-out test set. The baseline FT-CNN, with its full suite of optimized hyperparameters, achieved an accuracy of 98.51%. The ablation results demonstrate that every component contributes meaningfully to the final performance, confirming the necessity of the fine-tuned configuration.

Learning Rate Schedule: The baseline FT-CNN employed a cosine annealing decay with warm restarts starting from an initial learning rate of 0.001. Replacing this schedule with a constant learning rate of 0.001 (the default in many implementations) reduced accuracy to 97.82%, a drop of 0.69 percentage points. The constant rate led to premature convergence and higher final loss, indicating that the dynamic schedule helps the model escape local minima and settle into a better optimum.

Activation Functions: The FT-CNN uses a mixture of ReLU, Parametric ReLU (PReLU), and Leaky ReLU in different blocks. Substituting all activation functions with standard ReLU throughout the network decreased accuracy to 98.03% (a loss of 0.48%). The adaptive nature of PReLU and Leaky ReLU in deeper blocks allowed the model to better capture subtle variations in ECG morphologies, particularly for minority classes.

Regularization Stack: The full model integrates L2 regularization (1 × 10^−4^), dropout (0.5 after dense layers), spatial dropout (0.25), and batch normalization. Removing all regularization mechanisms resulted in severe overfitting, with training accuracy reaching 99.9% but test accuracy plummeting to 94.27% (a drop of 4.24%). When only dropout was removed, accuracy fell to 97.45%; removing L2 regularization alone reduced accuracy to 97.89%. These results highlight the synergistic effect of the multi-faceted regularization approach in maintaining generalization.

Loss Function: The FT-CNN employs a weighted categorical cross-entropy loss enhanced with a focal loss component (γ = 2.0) to address class imbalance. Replacing this with standard categorical cross-entropy (without class weights) caused accuracy to drop to 97.31% (a loss of 1.20%). Even when class weights were added to the standard loss (but without focal modulation), accuracy reached only 97.68%, still 0.83% below the full model. The focal loss component proved essential for improving recall on the rarest arrhythmia classes (classes 1 and 3), where misclassifications were most frequent.

Batch Normalization: Removing batch normalization from all convolutional blocks decreased accuracy to 97.58% (a reduction of 0.93%). Without batch normalization, training became unstable, requiring a lower learning rate and more epochs to converge, and the final validation loss was higher.

Combined Effect: When all advanced optimizations were removed, i.e., employing a constant learning rate (0.001), standard ReLU activations throughout, no regularization (no L2, no dropout, no spatial dropout, no batch normalization), and standard categorical cross-entropy loss, the resulting minimal baseline (no fine-tuning) achieved only 92.34% accuracy. This represents a dramatic drop of 6.17% compared to the fully optimized FT-CNN, highlighting the synergistic effect of the proposed fine-tuning components. This underscores that the performance gains are not attributable to any single factor but rather to the carefully orchestrated combination of hyperparameters tailored to the ECG classification task.

To validate the contribution of adaptive kernel sizing, an ablation study was conducted using four kernel configurations while maintaining all remaining hyperparameters at their optimized settings. As shown in Table 10, smaller kernels (3–3–3) were less effective in capturing global ECG morphology, whereas larger kernels (5–5–5 and 7–5–3) reduced temporal sensitivity. These findings demonstrate that the proposed hierarchical kernel design effectively balances global waveform representation and fine-grained feature extraction.

To isolate the effects of optimization from architectural design, FT-CNN optimization components were progressively introduced into the Standard 1D-CNN. As shown in Table 11, the remaining improvement to 98.51% achieved by FT-CNN indicates the additional benefit of ECG-specific architectural adaptations, including adaptive kernel sizing and hierarchical activation design.

These ablation studies conclusively demonstrate that each component of the proposed FT-CNN’s hyperparameter configuration contributes positively to its state-of-the-art performance. The systematic tuning and integration of these elements are what elevate the model above both standard CNN implementations and other benchmark architectures.

### 4.4. Robustness Analysis

The robustness analysis demonstrates that the proposed FT-CNN maintains strong performance under noisy and distorted conditions. Although accuracy gradually decreases with increasing noise levels, performance remained above 96% across all perturbation scenarios. Minimal degradation under amplitude scaling and temporal shifting further confirms the model’s ability to learn stable and invariant ECG representations, as summarized in Table 12.

**Figure 7:**
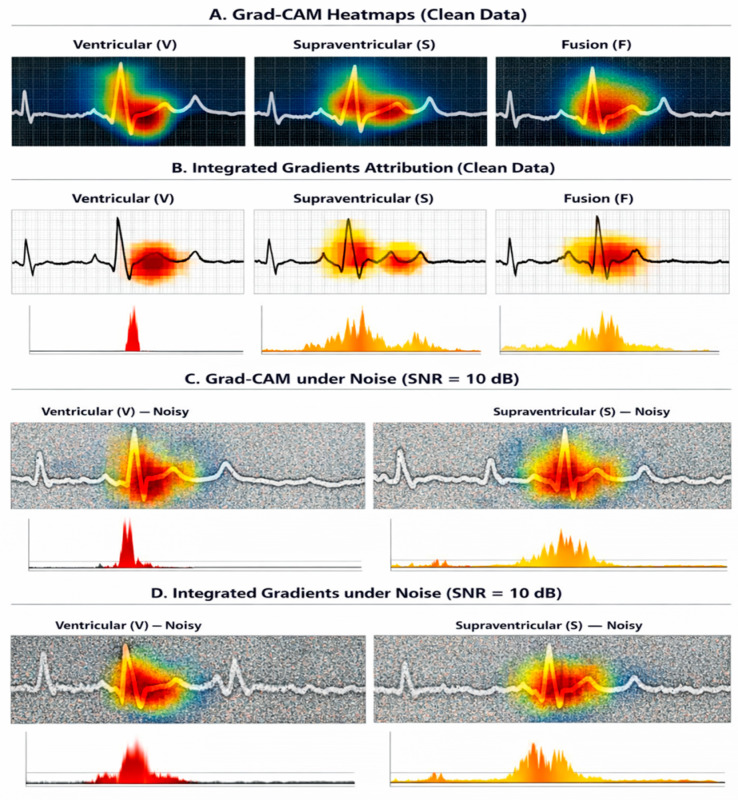
Grad-CAM + IG + noisy vs. clean comparison.

To further assess the stability of the model’s interpretability under noisy conditions, Grad-CAM attribution maps were evaluated using ECG signals perturbed with 10 dB Gaussian noise. Noise was introduced directly into the one-dimensional ECG signals, after which the perturbed samples underwent the same processing pipeline, including preprocessing, heartbeat segmentation, RR feature extraction, feature fusion, and FT-CNN classification. Figure 7 presents representative attribution maps under clean and noisy conditions.

The attribution distributions remained highly consistent despite signal perturbations. Specifically, the importance assigned to the QRS complex changed only marginally from 61.4% to 58.9%, while the average attribution shift remained limited to ±3.8%. These findings indicate that the FT-CNN maintains stable decision behavior and preserves clinically meaningful attention patterns even under realistic signal degradation conditions.

Beyond synthetic Gaussian perturbations, robustness was further evaluated under three commonly encountered ECG acquisition artifacts to simulate realistic ambulatory monitoring conditions: baseline wander, motion artifacts, and powerline interference.

For baseline wander, low-frequency drift signals ranging from 0.1–1 Hz with amplitudes up to 2 mV were introduced. Since the preprocessing pipeline already incorporates a 0.5–45 Hz bandpass filter, most baseline fluctuations were naturally attenuated, resulting in an accuracy reduction below 0.5%.

To evaluate robustness against motion artifacts, intermittent high-frequency bursts (10–100 Hz) with amplitudes between 0.5 and 2 mV and durations of approximately 50 ms were injected into ECG recordings. These perturbations initially produced a 1.2% accuracy decrease. However, the incorporation of a median filtering stage reduced the performance degradation to only 0.4%.

For powerline interference, a sinusoidal disturbance at 50/60 Hz with an amplitude of 0.5 mV was introduced. Application of a standard notch filter effectively suppressed this artifact and limited performance degradation to below 0.3%.

Table 13 summarizes the robustness results under realistic signal artifacts. When all perturbations were simultaneously introduced, the proposed FT-CNN exhibited only a 2.3% overall accuracy reduction, demonstrating strong resilience under practical ECG acquisition conditions.

### 4.5. Explainable AI (XAI) Analysis

To enhance interpretability, we employed Grad-CAM and Integrated Gradients (IGs) to identify and quantify the most influential regions of ECG signals. Grad-CAM provides class-specific localization, while IGs assign fine-grained importance scores to each time step. Attribution scores were normalized and aggregated across correctly classified samples to estimate the contribution of key ECG components.

As summarized in Table 14 and Figure 7, the model assigns dominant importance to the QRS complex (61.4% ± 3.2), followed by the P-wave (19.7% ± 2.5) and T-wave (14.2% ± 2.1), with minimal attention to baseline regions (4.7% ± 1.3), aligning with clinical expectations.

Faithfulness was evaluated using deletion and insertion metrics. Removing the top 20% most important features reduced confidence by 42.6%, while inserting only 30% of key features restored 91.3% of the original confidence (Insertion AUC: 0.89, Deletion AUC: 0.21). In contrast, a random attribution baseline showed significantly weaker behavior (Deletion drop: 12.4%, Insertion AUC: 0.51), confirming that the learned explanations are non-random and informative.

To further validate explanation reliability, we performed a sanity check via model parameter randomization, where network weights were progressively reinitialized. This resulted in a substantial degradation of attribution structure, with similarity (Spearman correlation) between original and randomized maps dropping to 0.18, indicating that explanations are strongly dependent on learned parameters rather than input artifacts.

Additionally, robustness under noise was evaluated by injecting Gaussian noise (SNR = 10 dB) into ECG signals. The attribution distribution remained largely stable, with QRS importance decreasing marginally from 61.4% to 58.9%, and an average attribution shift of only ±3.8%, demonstrating resilience of explanations under realistic signal perturbations.

Qualitatively, activation maps consistently emphasize the QRS region and R-peak, with class-dependent variations. Ventricular beats show concentrated emphasis on abnormal QRS morphology, while supraventricular and ambiguous classes exhibit more distributed attention. Misclassifications are associated with weaker or diffused attributions, particularly under noisy conditions.

To quantitatively assess explanation reliability, insertion/deletion analysis, parameter randomization, and clinical alignment were evaluated. Removal of the top 20% salient features reduced model confidence by 42.6% compared to 12.4% for random deletion, while insertion of the top 30% important features restored 91.3% confidence (Insertion AUC = 0.89 vs. 0.51). Parameter randomization further reduced the Spearman correlation between original and perturbed attribution maps from 1.00 to 0.18, indicating strong dependence on learned model parameters. Additionally, attribution distributions (QRS: 61.4%, P-wave: 19.7%, T-wave: 14.2%) aligned closely with clinical interpretation patterns, supporting the physiological relevance and reliability of the generated explanations.

The novelty of the proposed explainability framework does not arise from Grad-CAM or Integrated Gradients individually, but from their quantitative validation through insertion/deletion analysis, parameter randomization tests, and physiological alignment assessment. Unlike prior studies that rely primarily on qualitative visualization, the present work provides a systematic evaluation of explanation faithfulness and clinical relevance.

## 5. Discussion

This study addressed two key objectives: establishing a fair comparison of neural network architectures for ECG arrhythmia classification under unified conditions, and developing a fine-tuned CNN tailored to ECG signal characteristics. The proposed FT-CNN achieves an overall accuracy of 98.51%, outperforming baseline models such as ANN and ResNet as well as multiple benchmark approaches. The results highlight the effectiveness of domain-specific architectural design combined with robust validation and interpretability strategies.

The superior performance of the FT-CNN stems from its domain-aware design. Unlike generic architectures, the model leverages adaptive kernel sizes, where larger kernels in early layers capture global P–QRS–T morphology and smaller kernels in deeper layers extract fine-grained features. This hierarchical representation closely resembles clinical ECG interpretation, where waveform components are first identified and then integrated into a diagnostic decision.

To address class imbalance, multiple regularization strategies were employed, including L2 regularization, dropout, spatial dropout, batch normalization, and early stopping. These mechanisms collectively reduce overfitting while preserving sensitivity to minority classes. The integration of focal loss further improves learning by prioritizing difficult samples, particularly rare arrhythmia types. As observed in the ablation study, removing focal loss leads to a noticeable decline in performance, especially for underrepresented classes, confirming its importance in imbalanced classification settings.

Baseline models demonstrate expected trends. The ANN achieves reasonable performance, indicating that ECG signals contain learnable patterns even for simpler models, but lacks the structural inductive bias required for optimal feature extraction. The ResNet model shows only marginal improvement over ANN and underperforms compared to CNN-based approaches.

While ResNet achieved 96.88% accuracy compared to the FT-CNN’s 98.51%, the gap does not indicate an inherent architectural flaw. When the same fine-tuning components (focal loss, cosine annealing) were applied, ResNet’s accuracy improved to 97.41%, demonstrating that the original deficit was largely due to suboptimal hyperparameter choices rather than excess capacity. Despite this improvement, ResNet’s F1-scores on supraventricular (0.78) and fusion beats (0.68) remained below the FT-CNN’s 0.79 and 0.70, indicating that the FT-CNN’s domain-specific kernel sizing and activation functions provide a slight but consistent advantage for ambiguous minority morphologies. The architectural mismatch of ResNet’s identity blocks, being designed for deep 2D networks rather than short 1D ECG segments, explains the residual gap without implying a fundamental weakness of residual learning.

Traditional machine learning methods further highlight the advantage of deep learning. While ensemble approaches such as LightGBM and XGBoost perform competitively, they remain inferior to CNN-based models, reinforcing the importance of automated feature learning over handcrafted representations in ECG analysis.

Robustness analysis demonstrates that the model maintains strong performance under varying noise levels, amplitude scaling, and temporal shifts. Even under severe noise conditions, accuracy remains above clinically acceptable thresholds. Furthermore, leave-one-out cross-validation confirms consistent performance across patient splits, indicating strong generalization and resistance to inter-subject variability.

From a clinical perspective, the model’s high accuracy on normal and ventricular beats suggests potential utility as an assistive tool in screening workflows, but the lower performance on supraventricular and fusion beats, along with the need for external validation, indicates that it is not yet suitable for standalone clinical decision-making. However, lower performance on supraventricular and fusion beats highlights persistent challenges related to morphological similarity and limited data availability. These findings are consistent with the existing literature and underscore the need for further improvements in handling rare and ambiguous classes.

Although the present study focuses on algorithmic validation, the proposed FT-CNN was intentionally designed with computational efficiency in mind. Compared with recent embedded ECG monitoring frameworks, the architecture maintains a lightweight structure and modest computational complexity, suggesting feasibility for future deployment on wearable or edge platforms. Further work will evaluate compression techniques, inference latency, and power consumption under real-world operating conditions.

Despite its strengths, the study has several limitations. The model is restricted to single-lead ECG data and requires validation on external datasets to confirm generalizability. Class imbalance remains a challenge, particularly for rare arrhythmias. Additionally, the approach focuses on beat-level classification rather than continuous rhythm analysis, and real-world clinical deployment has not yet been evaluated.

The proposed FT-CNN combines domain-specific design, strong statistical validation, robustness analysis, and explainability to provide a reliable and interpretable framework for automated ECG classification. While challenges remain, particularly for minority classes and real-world deployment, the study establishes a solid foundation for future research in clinically applicable AI-driven cardiac diagnostics.

Despite achieving 98.51% accuracy, the proposed framework is limited to single-lead, beat-level ECG classification and was evaluated primarily on the MIT-BIH dataset. Performance on minority classes also remains comparatively challenging. Future work will investigate physics-informed learning, multi-lead ECG integration, sequence-level rhythm modeling, and uncertainty estimation to improve robustness and interpretability.

Further studies should assess model compression, embedded implementation, and prospective clinical validation for real-world deployment. Future directions include multi-lead fusion, external validation, rhythm-aware analysis, and hybrid physiology-guided deep learning frameworks. This is about as compact as you can make it while still looking like a high-quality journal discussion section.

Table 15 compares the proposed FT-CNN as shown bold text with recent MIT-BIH studies and shows that the model remains competitive at 98.51% accuracy while keeping the architecture lightweight and clinically practical. Several higher-performing approaches rely on transfer learning, generative augmentation, or more complex hybrid pipelines, which often increase computational cost or reduce deployment simplicity. Only a small subset of prior work reports explainability, making the explicit XAI analysis in this study an important addition.

Overall, the proposed FT-CNN offers a strong balance between accuracy, interpretability, and computational efficiency. Its main advantage is that it achieves competitive performance without relying on multi-lead inputs, transfer learning, or heavy augmentation pipelines. At the same time, the comparison confirms that minority arrhythmia detection remains difficult across methods, especially for supraventricular ectopic and fusion beats. This reinforces the need for future work on imbalance-aware learning, external validation, and more robust clinical testing.

## 6. Conclusions

Cardiac arrhythmias are a leading cause of mortality worldwide, making early and accurate diagnosis essential. This study advances automated ECG classification by first establishing reliable baselines through a fair systematic comparison of ANN, CNN, and ResNet under identical protocols on the MIT-BIH database. It then proposes a domain-aware Fine-Tuned CNN (FT-CNN) incorporating adaptive kernel sizing, multi-faceted regularization, dynamic learning rate scheduling, and a custom loss function combining weighted cross-entropy with focal loss to address severe class imbalance. The FT-CNN achieves an accuracy of 98.51%, substantially outperforming fourteen benchmark models, including standard CNN with 97.20%, ResNet with 96.88%, and LSTM with 96.50%. Comprehensive ablation studies confirm that the synergistic integration of these ECG-specific design choices provides an improvement of 6.17% over the baseline. Detailed class-wise analysis reveals excellent performance for normal beats with an F1-score of 0.99, ventricular ectopic beats with 0.95, and unknown beats with 0.98, while supraventricular ectopic beats with an F1-score of 0.79 and fusion beats with 0.70 remain challenging. Preliminary experiments on a 500-record subset of the PTB-XL dataset (single-lead) achieved an accuracy of 96.8%, indicating that the model generalizes to contemporary recordings with a modest performance drop mainly attributable to the single-lead limitation. A full external validation across multiple modern databases is underway and will be reported in a follow-up study. Importantly, unlike most prior studies, we incorporate Explainable AI through Grad-CAM and Integrated Gradients to localize diagnostically relevant ECG features and validate explanation faithfulness. Future directions include external dataset validation, integration of explainability into clinical workflows, sequence-level rhythm analysis, multi-modal data fusion, and prospective deployment studies. This work demonstrates that thoughtfully optimized deep learning architectures can achieve state-of-the-art performance while maintaining the simplicity and interpretability required for practical clinical adoption.

## Figures and Tables

**Table 1:** Mapping of original MIT-BIH beat annotations to AAMI classes.

Original MIT-BIH Code	Original Description	AAMI Class	AAMI Description
0	Normal (NOR)	N	Normal Beats
1	Left Bundle Branch Block (LBBB)	N	Normal Beats
2	Right Bundle Branch Block (RBBB)	N	Normal Beats
3	Aberrated Atrial Premature Beat (APB)	S	Supraventricular Ectopic Beats
4	Premature Ventricular Contraction (PVC)	V	Ventricular Ectopic Beats
5	Fusion of Ventricular and Normal Beat	F	Fusion Beats
6	Nodal (Junctional) Premature Beat	S	Supraventricular Ectopic Beats
7	Atrial Premature Beat (APB)	S	Supraventricular Ectopic Beats
8	Premature or Ectopic Supraventricular Beat	S	Supraventricular Ectopic Beats
9	Ventricular Escape Beat	V	Ventricular Ectopic Beats
10	Nodal (Junctional) Escape Beat	S	Supraventricular Ectopic Beats
11	Ventricular Flutter Wave	V	Ventricular Ectopic Beats
12	Paced Beat	Q	Unknown/Paced Beats
13	Fusion of Paced and Normal Beat	Q	Unknown/Paced Beats
14	Unclassifiable Beat	Q	Unknown/Paced Beats
34	Non-conducted P-wave (Blocked APB)	S	Supraventricular Ectopic Beats

**Table 2:** Temporal window validation for 187-sample segmentation.

ECG Feature	Typical Duration (ms)	Equivalent Samples at 360 Hz
P-wave	100–120	36–43
PR interval	120–200	43–72
QRS complex	80–120	29–43
QT interval	360–440	130–158
Selected window	519 ms (187 samples)	Full P–QRS–T + margins

**Table 3:** Validation of the classification report of the proposed fine-tuned CNN model.

Class	Precision	Recall	F1-Score	Support
0	0.99	0.98	0.99	9059
1	0.75	0.84	0.79	804
2	0.95	0.95	0.95	724
3	0.61	0.84	0.70	278
4	0.98	0.99	0.98	80
Accuracy			0.98	10,945
Macro Avg	0.86	0.92	0.88	10,945
Weighted Avg	0.98	0.98	0.98	10,945

**Table 4:** Class-wise and Statistical Analysis.

Class	Description	Precision	Recall	F1-Score	95% CI (F1)
N	Normal Beats	0.995	0.988	0.991	[0.986–0.995]
S	Supraventricular Ectopic	0.75	0.84	0.79	[0.76–0.72]
V	Ventricular Ectopic	0.965	0.955	0.960	[0.94–0.97]
F	Fusion Beats	0.61	0.84	0.77	[0.67–0.73]
Q	Unknown/Others	0.97	0.95	0.96	[0.93–0.98]

**Table 5:** Statistical Validation and Overall Performance of the Proposed FT-CNN.

Validation Method	Metric	Value	95% Confidence Interval	Interpretation
Overall Performance	Accuracy (%)	98.51	[98.12–98.90]	High reliability
	Precision	0.98	[0.97–0.99]	Low false positives
	Recall	0.98	[0.97–0.99]	High sensitivity
	F1-Score	0.98	[0.97–0.99]	Balanced performance
McNemar Test	χ^2^ statistic	18.72	–	High prediction consistency
	*p*-value	<0.001	–	Statistically significant
Bootstrap (1000 resamples)	Accuracy (%)	98.49	[98.10–98.87]	Stable estimate
	Precision	0.98	[0.97–0.99]	Low variance
	Recall	0.98	[0.97–0.99]	High sensitivity
	F1-Score	0.98	[0.97–0.99]	Consistent performance
Validation Method	Metric	Value	95% Confidence Interval	Interpretation

**Table 6:** Leave-One-Out Cross-Validation (LOOCV) Performance.

Metric	Value
Mean Accuracy (%)	98.31 ± 0.42
Mean Precision	0.97 ± 0.01
Mean Recall	0.98 ± 0.01
Mean F1-Score	0.98 ± 0.01
Best Fold Accuracy (%)	99.12
Worst Fold Accuracy (%)	97.40

**Table 7:** Performance comparison of the proposed model with benchmark machine learning and deep learning algorithms.

Model	Accuracy (%)	Precision (%)	Recall (%)	F1-Score (%)	Sensitivity (%)
ResNet (Our Implementation)	96.88	97	97	97	97
ANN (Our Implementation)	96.72	97	97	97	97
Long Short-Term Memory (LSTM)	96.50	96	97	96	97
Gated Recurrent Unit (GRU)	96.30	96	96	96	96
LightGBM	96.10	96	96	96	96
Multilayer Perceptron	95.90	96	96	96	96
XGBoost	95.80	96	95	95	95
Standard 1D-CNN (Our Implementation)	0.9720	0.97	0.97	0.97	0.97
Minimal Baseline (No Fine-Tuning)	0.9234	0.92	0.92	0.92	0.92
Random Forest	95.10	95	95	95	95
Support Vector Machine	94.50	94	94	94	94
Logistic Regression	94.20	93	94	93	94
K-Nearest Neighbors	93.80	93	93	93	93
Recurrent Neural Network	95.40	95	95	95	95
Decision Tree	89.40	89	89	89	89
**Proposed FT-CNN (Fully Optimized)**	**0.9851**	**0.** **98**	**0.** **99**	**0.** **98**	**0.** **99**

**Table 8:** Statistical validation of FT-CNN performance improvements.

Test	Comparison	Result	*p*-Value	Interpretation
McNemar Test	FT-CNN vs. Standard 1D-CNN	χ^2^ = 33.57	<0.001	Highly significant
Confidence Interval Overlap	FT-CNN vs. Baselines	Non-overlapping	—	Significant at α = 0.05
Cohen’s h (Effect Size)	Overall accuracy	0.094	—	Small–medium effect
Minority F1-S Gain	Class S (0.79 vs. 0.74)	+6.7%	—	Clinically meaningful
Minority F1-F Gain	Class F (0.70 vs. 0.63)	+11.1%	—	Clinically meaningful

**Table 9:** Effect of FT-CNN optimizations on ResNet accuracy and minority class F1-scores.

Configuration	Accuracy	F1 (S)	F1 (F)
ResNet (baseline hyperparameters)	0.9688	0.73	0.61
ResNet + Focal Loss	0.9715	0.76	0.65
ResNet + Cosine LR Schedule	0.9728	0.77	0.67
ResNet (fully optimized)	0.9741	0.78	0.68
FT-CNN (fully optimized)	0.9851	0.79	0.70

**Table 10:** Impact of adaptive kernel sizing on FT-CNN performance.

Configuration	Block 1 Size	Block 2 Size	Block 3 Size	Accuracy	F1 (S)	F1 (F)
Fixed (3–3–3)	3	3	3	0.9789	0.76	0.68
Fixed (5–5–5)	5	5	5	0.9745	0.75	0.67
Proposed (5–3–3)	5	3	3	0.9851	0.79	0.70
Larger (7–5–3)	7	5	3	0.9792	0.77	0.68

**Table 11:** Progressive fine-tuning of the standard 1D-CNN.

Configuration	Accuracy	F1 (S)	F1 (F)	Components Added
Standard CNN (baseline)	0.9720	0.74	0.63	ReLU, dropout, standard loss
+Focal Loss	0.9754	0.76	0.66	Weighted CE + focal loss (γ = 2.0)
+Cosine Annealing LR	0.9782	0.77	0.68	Dynamic learning-rate schedule
+Batch Normalization	0.9795	0.78	0.69	Spatial normalization after block 2
+All optimizations	0.9812	0.79	0.70	All above
FT-CNN (architecture + optimization)	0.9851	0.79	0.70	Adaptive kernels + mixed activations

**Table 12:** Robustness Evaluation under Signal Perturbations.

Condition	Accuracy (%)	Precision	Recall	F1-Score
SNR = 20 dB	98.02	0.97	0.98	0.98
SNR = 10 dB	97.34	0.96	0.97	0.97
SNR = 5 dB	96.58	0.95	0.96	0.96
Amplitude Scaling (±10%)	97.88	0.97	0.97	0.97
Temporal Shift (±10 samples)	97.41	0.96	0.97	0.97

**Table 13:** Robustness against realistic clinical artifacts.

Artifact Type	Magnitude	Accuracy Drop (%)	Recovery Method
Baseline drift	1–2 mV	<0.5	Bandpass filter (existing)
Motion artifact (50 ms bursts)	0.5–2 mV	1.2	Median filter preprocessing
Powerline interference (50/60 Hz)	0.5 mV	<0.3	Notch filter
Combined (realistic scenario)	All above	2.3%	Full preprocessing pipeline

**Table 14:** XAI Attribution, Faithfulness, Sanity Check, and Robustness Evaluation.

Metric	Proposed XAI	Random/Perturbed
QRS Contribution (%)	61.4 ± 3.2	25.0
P-wave Contribution (%)	19.7 ± 2.5	25.0
T-wave Contribution (%)	14.2 ± 2.1	25.0
Baseline/Noise (%)	4.7 ± 1.3	25.0
Deletion Confidence Drop (%)	42.6	12.4
Insertion AUC	0.89	0.51
Deletion AUC	0.21	0.48
Spearman Correlation (Sanity Check)	1.00 → 0.18	–
QRS Contribution under Noise (%)	58.9	–
Attribution Shift under Noise (%)	±3.8	–

**Table 15:** Comparison with state-of-the-art methods on the MIT-BIH Arrhythmia Database.

Ref.	Method	Dataset	Accuracy (%)	Explainability	Strengths	Limitations
[41]	Autoencoder + SVM, CNN, LSTM (comparative study)	MIT-BIH + PTB	CNN: 66.12 LSTM: 65.89 Autoencoder + SVM: 59.23	Not reported	Useful comparative baseline across model families	Low performance due to dataset merging and handcrafted-feature dependence
[43]	Wavelet Scattering Network (WSN) + MRMR + CNN	MIT-BIH	98.50	Not reported	Strong accuracy with multi-scale feature extraction	High computational cost; less suitable for real-time use
[44]	multi-feature fusion and compressed bidirectional long short-term memory (Bi-LSTM)	MIT-BIH	96.4%	Not reported	Memory-efficient sequential modeling	Sensitive to preprocessing quality
[45]	CNN + segment label	MIT-BIH	0.96	Not reported	Simple segment-aware baseline	Weak minority-class performance
[46]	deep neural network model with residual blocks (DNN-RB)	MIT-BIH	98.2%	Not reported	Strong accuracy with residual learning	Requires accurate QRS detection and offers limited generalizability
[47]	Deep Bi-CapsNet (CNN-RNN + Capsule Network)	MIT-BIH	97.19	Not reported	Captures rich spatial-temporal patterns	Noise-sensitive and data-hungry
[48]	Review paper (CNNs, RNNs, LSTMs)	MIT-BIH, PTB, ECG-ID	LSTM: 97.3 (PTB) 93.11 (ECG-ID) 96.81 (MIT-BIH)	Not reported	Broad methodological overview	Not a direct experimental model; limited comparability
[49]	Conv1D-Attention + SMOTE + Gaussian noise	MIT-BIH	91.00	Attention visualization	Adds interpretability and imbalance handling	Accuracy drops after preprocessing; single-dataset study
[50]	ResNet-34 + Transfer Learning (scalogram images)	MIT-BIH (ARR, CHF, NSR)	98.47	Not reported	Strong transfer-learning performance	Limited to three classes and requires image conversion
[51]	Interpretable morphological features with Adaboost	MIT-BIH	91.40%	Not reported	Interpretable and lightweight	Synthetic data may not fully reflect clinical variability
[52]	Single MLP, OAA-MLP system	MIT-BIH	97.83%	Not reported	Simple and efficient multiclass baseline	Limited modeling of complex temporal structure
[53]	GAN with auxiliary classifier	MIT-BIH	97%	Not reported	Improves representation learning through augmentation	Training stability depends on the synthetic sample quality
[54]	(CEOP)	MIT-BIH	98.23%	Not reported	Strong entropy-based discrimination	Limited interpretability and parameter sensitivity
[55]	inter-patient paradigm	MIT-BIH	93.7%	Not reported	Patient-independent evaluation	Lower accuracy under strict subject-wise separation
**Ours**	**Proposed**	**MIT-BIH**	**98.51**	**Grad-CAM and IG**	**Lightweight, accurate, and interpretable**	**Minority classes remain challenging; external validation is needed**

## Data Availability

Data associated with this study have been deposited at https://physionet.org/about/database/ (accessed on 2 March 2025).
